# Modified dose intensive R- CODOX-M/IVAC for HIV-associated burkitt (BL) (AMC 048) shows efficacy and tolerability, and predictive potential of IRF4/MUM1 expression

**DOI:** 10.1186/1750-9378-7-S1-O14

**Published:** 2012-04-19

**Authors:** Ariela Noy, Lawrence Kaplan, Jeannette Lee, Ethel Cesarman, Wayne Tam

**Affiliations:** 1 Memorial Sloan-Kettering Cancer Center, New York, NY, USA; 2 University of California, San Francisco, San Francisco, CA, USA; 3 University of Arkansas for Medical Sciences, Little Rock, Little Rock, AR, USA; 4 Weill Cornell Medical College and New York Presbyterian Hospital, New York, NY, USA

## Background

HIV associated BL remains of concern for toxicity of dose-intensive regimens used in HIV negative patients (pts). Less intensive regimens have a high relapse rate. We modified CODOX-M/IVAC hoping to preserve efficacy while improving tolerability, particularly treatment related mortality (TRM). Primary object: improving 1 year overall survival (OS) from the historical 65 to 85%.

## Methods

Modifications of the US NCI regimen include rituximab (R), cyclophosphamide reduction [800 mg/m2 x 2 days], vincristine 2 mg cap, methotrexate (mtx) 3000 mg/m2, dual chemotherapy lumbar punctures and IVAC infusion (high risk pts). Antibiotic prophylaxis & growth factor support specified, 100% grade IV hematopoietic toxicities in the original regimen. HAART therapy at the discretion of the local MD. Pathology review included CD20, CD10, BCL2, BCL6, p53, Ki67, BLIMP1, IRF4/MUM1 and EBV EBER. (Table 1)

**Table 1:** Table 1

Status	N (%)
Treatment Completed per protocol	21 (62%)
Disease Progression	3 (9%)
Early termination due to adverse event*	5 (15%)
Early termination due to patient withdrawal**	2 (6%)
Early termination – counts did not recover within time frame to begin cycle 4	1 (3%)
Treatment ongoing	2 (6%)

*1 pt with grade (gr) 4 thrombocytopenia and gr 3 infection; 1 pt with gr 3 left hemiparesis; 1 pt with gr 3 confusion unrelated to treatment; 1 pt with prior hepatitis B and cirrohosis had gr 3 encephalopathy and pulmonary infiltrates; 1 pt with gr 4 neutropenia and gr4 thrombocytopenia.

**1 CR 2 yrs post treatment.

## Results

Accrual of 33 planned pts by April 2010. Baseline: Classical Burkitt, 97%; Low/High Risk, 9/91%; Median (range) Age 42 (19 – 55); CD4 count 195 (0 - 721), CD4 <100, ^5^ (27%); HIV viral load 1819 (Undetectable – 1,187,968). Median follow up (fu) is 9 mos for surviving pt. Number of pts with gr3/4 toxicity: any 20 (61%), 13 (39%) hematologic, 16 (48%) infection including 7 febrile neutropenia, 6 metabolic with 1 tumor lysis syndrome, 4 neurologic, 2 thrombotic and 1 each coagulation, GI or pain. Only 2 gr 1/2 stomatitis/mucositis; 0 had gr 3/4. Six deaths: encephalopathy with hepatic failure, hepatitis B and pneumonia (1), disease progression (3) including 1 in the CNS; fungal infection (1); HIV. Median 1 year OS (n=34) was 81.7% (61.0%, 92.1%) with a 35 mo median survival. OS by non-BL defining proteins: EBER +/- (8/16) and p53 +/- (10/10) were not predictive. IRF4/MUM1 +/- (8/15) highly predictive in overall pts, but not in the confirmed Burkitt +/- (6/14) with only 1 IRF4/MUM1 neg pt dying of BL.

## Conclusions

AMC 048 with a median fu of 9 mos has a 1 yr OS of 82% in BL. Relapses after 1 year are rare. TRM was zero. R did not appear to increase toxicity. Only 5 pts withdrew due to AEs. Grade 3/4 toxicities were markedly reduced. Results compare favorably with 2 studies of HIV neg pts. Magrath (1995) reported 100% grade 4 hematologic and 20% grade 4 mucositis in 39 adults, 33 children (92% 2 yr EFS). MRC/NCRI LY10 trial (Mead 2008) reduced mtx (3gr/m2), but reported 9% TRM (64% 2 yr OS). IRF4/MUM1 deserves further study in BL.

